# Neurochemical Architecture of the Central Complex Related to Its Function in the Control of Grasshopper Acoustic Communication

**DOI:** 10.1371/journal.pone.0025613

**Published:** 2011-09-28

**Authors:** Michael Kunst, Ramona Pförtner, Katja Aschenbrenner, Ralf Heinrich

**Affiliations:** Department of Cellular Neurobiology, Institute for Zoology, University of Göttingen, Göttingen, Germany; Max-Planck Institute of Neurobiology, Germany

## Abstract

The central complex selects and coordinates the species- and situation-specific song production in acoustically communicating grasshoppers. Control of sound production is mediated by several neurotransmitters and modulators, their receptors and intracellular signaling pathways. It has previously been shown that muscarinic cholinergic excitation in the central complex promotes sound production whereas both GABA and nitric oxide/cyclic GMP signaling suppress its performance. The present immunocytochemical and pharmacological study investigates the question whether GABA and nitric oxide mediate inhibition of sound production independently. Muscarinic ACh receptors are expressed by columnar output neurons of the central complex that innervate the lower division of the central body and terminate in the lateral accessory lobes. GABAergic tangential neurons that innervate the lower division of the central body arborize in close proximity of columnar neurons and thus may directly inhibit these central complex output neurons. A subset of these GABAergic tangential neurons accumulates cyclic GMP following the release of nitric oxide from neurites in the upper division of the central body. While sound production stimulated by muscarine injection into the central complex is suppressed by co-application of sodium nitroprusside, picrotoxin-stimulated singing was not affected by co-application of this nitric oxide donor, indicating that nitric oxide mediated inhibition requires functional GABA signaling. Hence, grasshopper sound production is controlled by processing of information in the lower division of the central body which is subject to modulation by nitric oxide released from neurons in the upper division.

## Introduction

Reproductive and agonistic behaviors of acoustically communicating grasshoppers include the production of species- and situation-specific sound patterns. Grasshoppers generate acoustic signals for mate attraction, courtship and rivalry by rhythmically rubbing their hindlegs against the forewings ( = stridulation) (reviewed by [1]). The neuromuscular excitation patterns for sound generating hind leg movements are generated by central pattern generators in the metathoracic ganglion which are connected to the brain via sets of descending stridulatory command neurons [2], [3]. Each of several types of these command neurons activates only one stridulatory pattern [4]. Song pattern generation in thoracic ganglia and its control by neural circuits in the brain are anatomically separated and can be studied independently.

Initiation of song production and selection of song patterns is mediated by brain neuropils, most importantly by the central complex. Pharmaco-behavioral studies on the grasshopper *Chorthippus biguttulus* identified a number of different neurotransmitters and second-messenger pathways that activate or suppress sound production upon focal injection into the central complex [5]–[8], [9], [10]. Single injections of cholinergic agonists into the central complex elicited courtship song sequences that contained various different patterns in the correct natural sequence suggesting that central complex circuits select and coordinate the composition of song patterns and time the activity of the respective command neurons [5], [7].

In restrained but otherwise intact male and female grasshoppers, stridulation can be stimulated by injection of ACh and both its nicotinic and muscarinic agonists into their central body [5], [7]–[9]. In contrast, GABA and NO, via activation of cGMP-signaling pathway, suppressed muscarine-stimulated sound production [6], [10]. In addition to cholinergic excitation, sound production can also be elicited by disinhibition resulting from pharmacological inhibition of NO-formation or chloride channel-associated receptors [6], [11], indicating that stridulation is controlled by a balance of excitation and inhibition in the central complex. Cholinergic excitation and NO-mediated inhibition in the central complex have been linked to sensory signals or behavioral situations that either promote (e.g. hearing the song of a conspecific) or suppress (e.g. handling, restraint) sound production and both, muscarinic excitation and inhibition of NO-formation increased the responsiveness of grasshoppers to conspecific songs [8], [11], [12].

**Figure 1:**
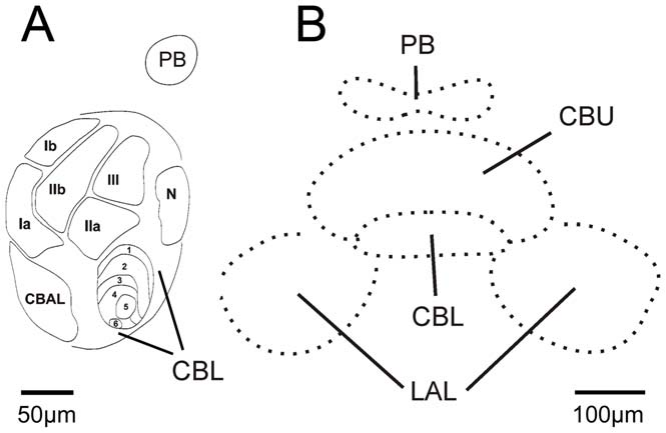
Neuropils of the grasshopper central complex. A: Sagittal section through the upper (CBU) and lower (CBL) division of the central body and one nodulus (N). Left side of the drawing is anterior and top is dorsal with respect to the grasshopper's body axis. The central body includes fronto-horizontal layers, three layers (layer I–III) plus the central body anterior lip (CBAL) in the upper division and six in the lower division (layer 1–6). The layers I and II of the upper division can both be subdivided into compartments a and b (modified after [18], [21]. B: Frontal section through the protocerebral bridge (PB), the upper (CBU) and lower (CBL) division of the central body and the lateral accessory lobes (LAL). Top of the drawing is dorsal and bottom is ventral with respect to the grasshopper's body axis.

The central complex (CX) is a midline spanning network of highly structured neuropils in the center of the insect midbrain. It includes four interconnected subunits (Fig. 1): the protocerebral bridge (PB), the upper (CBU) and lower divisions (CBL) of the central body, and the paired noduli [13], [14], [15]. As known from studies on various insect species, the CX is arranged in defined layers (Fig. 1A) which are intersected by either eight or sixteen columns [13], [16]–[20]. This regular structure results from two classes of interneurons that innervate the central complex, tangential and columnar neurons. Tangential neurons provide input from the median protocerebrum (mainly from the lateral accessory lobes) to all columns connected by individual central body layers. Columnar neurons connect the columns of the protocerebral bridge and the central body upper and lower division in a regular pattern of ipsi- and contralateral projections and send information to the contralateral lateral accessory lobes (LAL), the major input/output neuropils of the CX [21], [22]. In addition, intrinsic neurons of the central body, the pontine neurons, connect different columns of the upper division.

In order to study the flow and processing of information within the central complex, we mapped the distribution of neurotransmitters (GABA), receptors (muscarinic ACh-receptors) and neural signaling-associated metabolites (cGMP, citrulline) that have been demonstrated to contribute to the cephalic control of stridulation in previous pharmacological studies. The results indicate, that suppression of sound production is mediated by GABA release from tangential neurons in the CBL, that seem to directly inhibit muscarinic receptor-expressing and stridulation activating columnar output neurons of the CX. In contrast, NO released in the CBU mediates its stridulation suppressing effect indirectly, via stimulation of cGMP accumulation in GABAergic terminals in the CBL. This hypothesis was supported by pharmacological experiments. The present study identifies the CBL as the neuropil where pathways that promote or suppress sound production converge on columnar output neurons of the central complex.

## Materials and Methods

### Animals

Adult specimen of the grasshopper *Chorthippus biguttulus* (L. 1758) were caught in public and non-protected areas in the vicinity of Göttingen, Germany, and kept separately in the laboratory for up to several weeks. *Ch. biguttulus* is a common species in middle Europe that is neither endangered nor protected. Additional *Ch. biguttulus* were reared from eggs that were collected in the previous summer and kept at 4°C for at least 4 months. The nymphs hatched after ∼1 week at 26°C and were raised to adulthood on wheat and supplemental food for crickets (Nekton Pforzheim) at a 16/8 h light dark cycle.

### Immunocytochemistry

The following primary antisera were used: rabbit anti-mAChR (1∶200, generous gift by D.B. Satelle [23]), guinea pig anti-GABA (1∶1000, Protos Biotech, New York), sheep anti-cGMP (1∶5000, generous gift by J. DeVente [24]) and mouse anti-citrulline (1∶20, generous gift by G.R. Holstein [25]). Animals were anesthetized by cooling to 4°C, decapitated and their brains dissected. Brain tissues were fixed over night at 4°C in 4% paraformaldehyde dissolved in 0.1 M phosphate buffer (PB). Brains were embedded in a mixture of albumine/gelatine, postfixed at 4°C in 4% PFA and sectioned with a vibratome (Leica Vibracut VT 1000) into slices of 30–50 µm. Sections were rinsed over night in 0.1 M phosphate buffered saline (PBS) containing 1% Triton X-100 (PBST). For citrulline immunostaining, brains were fixed in 4% PFA and 1% glutaraldehyde for 3 hours and incubated directly after sectioning for 10 minutes in 0.1 M sodiumborohydride (in PBS) to reduce glutaraldehyde-induced autofluorescence. Prior to incubation with primary antisera, sections were blocked in 0.25% BSA and 5% normal goat/donkey serum dissolved in PBST. Primary antisera were incubated at 4°C for 2 days on a rocking table. Sections were incubated over night at 4°C with the following secondary antibodies: goat anti-mouse Alexa 488, donkey anti-rabbit Alexa 555, goat anti-rabbit Alexa 633 (all diluted 1∶300), donkey anti-sheep Alexa 633 (1∶50) (all Molecular Probes), donkey anti-guinea pig Cy2 (1∶200) (Jackson Immunoresearch). For cGMP immunostaining, brains were incubated prior fixation in 10^−2^ M sodium nitroprusside (SNP, Sigma) and 5×10^−4^ 3-(5′hydroxymethyl-2′furyl)-1benzyl indazole (YC-1, Sigma), an activator of soluble guanylyl cyclase [26] dissolved in grasshopper saline to stimulate accumulation of cGMP via soluble guanylyl cyclase activation (for detailed protocol see [10]).

Sections were washed several times in PBST, transferred to a 1∶1 mixture of PBS and glycerol, and mounted on slides for microscopic analysis.

### Specificity controls

To label mAChRs on grasshopper brain sections, a polyclonal antiserum raised in rabbit against the mAChR of *Drosophila melanogaster* [23] was used that has been demonstrated by Western blotting to bind a protein of similar molecular mass (approx. 90 kDa) in *Ch. biguttulus* [12].

The guinea-pig antiserum (Protos Biotech, NT 108 GABAgp) was generated against GABA. To test specifity of the polyclonal guinea-pig GABA antiserum on brain sections of *Ch. biguttulus* liquid-phase preadsorption with various concentrations of GABA-BSA conjugates were performed. Immunostaining was abolished after preadsorption with 20 mg/ml GABA-BSA conjugate (Fig. S1).

The monoclonal anti-citrulline antiserum has been characterized by Martinelli et al. [25]. The mouse antiserum was raised against citrulline conjugated to keyhole limpet hemocyanin (KLH). Preadsorption of the antiserum with a citrulline-bovine serum albumin has previously been performed on the locust *Schistocerca gregaria* [27]. Conjugate concentrations of 10 nM abolished all subsequent immunostaining. In addition, injection of the NOS inhibitor aminoguanidine into the grasshopper central complex abolished subsequent citrulline immunostaining, confirming that the antibody specifically detected citrulline [11].

The specifity of the sheep anti-cGMP antiserum, which was raised against formaldehyde fixed cGMP, has been demonstrated by blotting different nucleosides on nitrocellulose membanes (for a detailed description of the specificity controls see [28] and the antiserum has previously been shown to detect cGMP in a variety of insect species [10], [29]–[32], among them *Ch. biguttulus*, the species used in this study. Preadsorption with 1 µg/ml of a BSA-cGMP conjugate eliminated all immunostaining in the grasshopper *S. gregaria* [27]. Cyclic GMP-like immunofluorescence exclusively appeared after preincubation of grasshopper brains with NO, YC-1 and phosphodiesterase inhibitors, confirming that the antibody specifically detects cGMP in *Ch. biguttulus*.

### Analysis of anatomical data

The terminology for brain structures follows Strausfeld [16]. Central complex subdivisions are named according to Homberg [15], [18] and Müller et al. [21]. Positional information is given with respect to the body axis of the animal. Images were obtained with a Leica confocal laser scanning microscope (TCS SP2). For colocalisation studies, specimen were imaged with a 40× oil immersion objective with a NA of 1.25. The voxel size was set to a optimal value (96×96×163 nm) according to the Nyquist theorem, meaning that the smallest resolvable unit was sampled twice. Subsequent image processing included adjustment of brightness and contrast and a background substraction with a rolling ball radius of 50 pixels. To reduce background noise a median filter with a kernel radius of 2 was applied in some cases. All images were processed with the ImageJ software (developed at the U.S. National Institutes of Health and available at http://rsb.info.nih.gov/ij/). Colocalisation was measured by a distance-based colocalistion analysis [33] using the JACoP-plugin. Images shown in the results part are single optical sections in which colocalised pixels are highlighted in white. Percentage values of colocalisation are calculated for the pixels through the entire z-dimension of the image stack.

### Pharmacology

Adult male grasshoppers were attached with wax to a holder. The dorsal part of the head capsule was opened with razor blade and its frontal part with the brain was tipped forward to expose the dorsal (referring to the neural axis) surface of the brain. Small pieces of reflecting foil (Scotchlite 3 M, type 7610) were glued to the femur of each hind leg to enable the recording of stridulatory movements with two optoelectronic devices. Voltage signals proportional to the elevation of each hind leg were digitized with a sampling rate of 3 kHz (A/D converter: Real Time Devices AD3300; software: Turbolab 4.3, Bressner Technology) and stored as data files on a regular PC. The software NEUROLAB [34] was used to determine the periods of song production. Muscarine, sodium nitroprusside and picrotoxin (all from Sigma Aldrich) were dissolved in grasshopper saline [35] to concentrations of 10^−3^ or 10^−4^ M. Drugs were applied through capillaries with two chambers pulled to a single tip. Coupling to a pressure pump (WPI model PV 820) enabled injections of small volumes (1–3 nl) from either chamber to the same site within the brain. Stimulating drugs were injected in regular intervals of 5 min to maintain a similar level of overall excitation. Inhibitory drugs were injected once (after the activity released by the stimulating drug was terminated) and their influence on subsequent injections of the stimulating drug evaluated.

### Analysis of pharmacological experiments

One injection of a stimulating drug (muscarine or picrotoxin) usually elicited several song sequences separated by short pauses. The sum of the durations of all song sequences released by one stimulus was taken as the total duration of stridulation and the highest value obtained in one experiment at a particular stimulation site was set as 100%. Relative total durations of song production of three stimuli preceding the application of the inhibitory drug were averaged and used as a reference. Potential changes in the duration of muscarine- or picrotoxin-stimulated song production following one injection of sodium nitroprusside were evaluated with the non-parametric Friedman test for multiple dependent values followed by the Wilcoxon-Wilcox test to identify the significantly different data sets. Calculations were made with Excel (version 9, Microsoft) and figures assembled with Photoshop (Adobe Systems).

## Results

### Columnar central complex neurons express muscarinic ACh receptors

**Figure 2:**
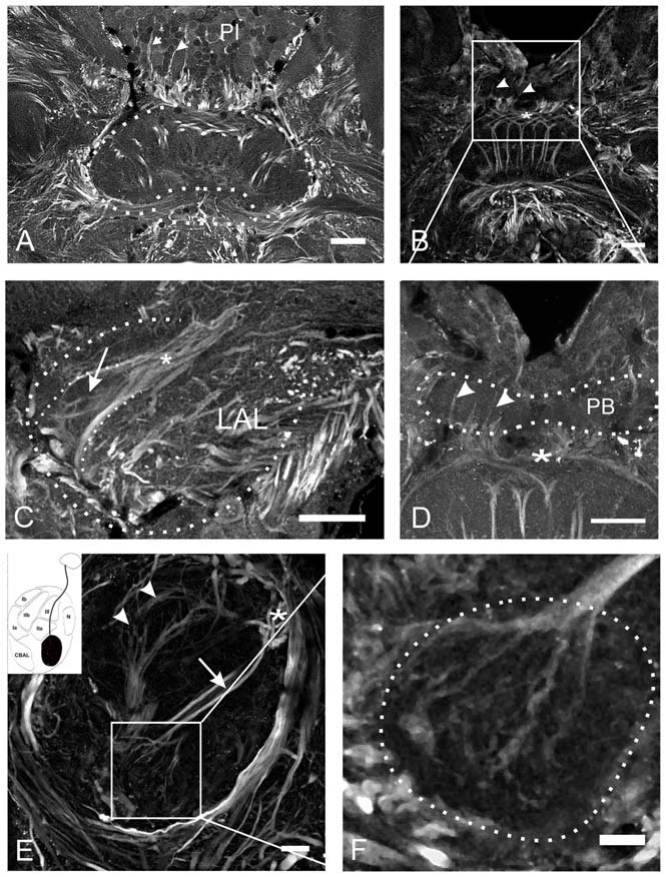
Muscarinic ACh receptor-immunoreactivity (mAChR-ir) in the central complex. A–D: Frontal sections of the central complex. MAChR-ir is restricted to columnar fibers whose somata are located in the pars intercerebralis (PI). These neurons send small neurites into the protocerebral bridge (PB) (arrowheads in A, B and D). The main fibers project as large diameter neurites via four pairs of fiber bundles, the w-, x-, y- and z-bundles through the posterior chiasm (indicated by white asterisk in A, B, D and E) between the protocerebral bridge and the central body and innervate single columns of the lower division. The fibers run to the contralateral lateral accessory lobe (LAL) via the isthmus tract (indicated by white asterisk in C) and seem to terminate in the lateral triangle (indicated by arrow in C). E: Sagittal section through the central body with two types of mAChR-ir columnar fibers. One type (indicated by arrowheads) runs through layer I of the CBU and passes along the anterior border of the CBL, while the other type projects through layer III as part of the posterior vertical bundle (indicated by arrow) and innervates the CBL (The inset describes the projection pattern of the second type; modified from [18], [21]. F: Sagittal section of the CBL. The arborization pattern in the lower division is not restricted to a certain layer but extends rather diffusely throughout the entire CBL. Smooth appearance of neurites indicates their post-synaptic character. Scale bars = 50 µm in A, B, C and D; 20 µm in E; 10 µm in F.

Muscarinic AChR immunoreactivity in the central complex (Fig. 2) was observed in fibers of two types of columnar neurons that connect the central complex with the lateral accessory lobes. The weakly stained somata of both types of neurons were located in the pars intercerebralis (PI, Fig. 2A) and mAChR expressing neurons gave rise to a number of thin neurites within the protocerebral bridge (arrowheads in Fig. 2A, B and D). Large diameter fibers projected via four pairs of fiber bundles, the w-, x-, y- and z-bundles [13] through the posterior chiasm (PCh, indicated as white asterisk in Fig. 2B, D and E), which is located between the protocerebral bridge and the central body. One fiber type was passing through layer I (indicated by arrowheads in Fig. 2E) of the upper division and projected dorsally along the anterior border of the CBL. As visualized in sagittal sections through the central complex, the other type of mAChR immunopositive fibers passed as part of the posterior vertical bundles (PVB, indicated by white arrow in Fig. 2E) through layer III of the central body upper division and formed arborizations that innervated the lower division with smooth endings (Fig. 2E, F). From the central body, the fibers were projecting to the contralateral LAL. Here fibers were only weakly stained, projected through the isthmus tract (asterisk in Fig. 2C) and seemed to innervate the lateral triangle of the LAL (arrow in Fig. 2C). Muscarinic AChR immunoreactivity in other regions of the *Ch. biguttulus* brain is shown in figure S2.

### GABA immunoreactivity in the central complex

**Figure 3:**
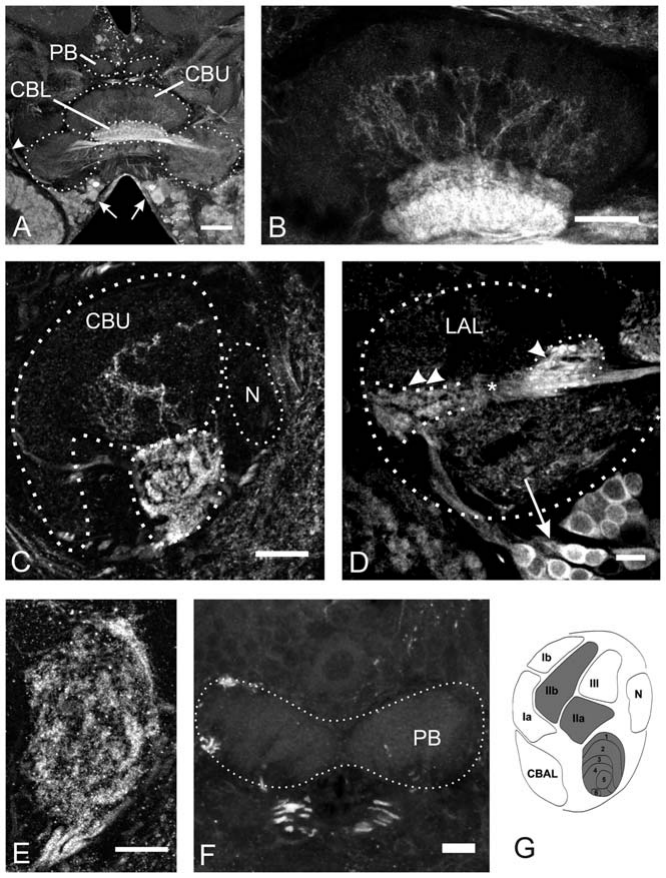
GABA immunoreactivity in the central complex. A and B: Frontal sections through the midbrain (A) and the central body (B). The entire lower division (CBL) is densely innervated by GABA positive neurites, while only few neurites in the upper division (CBU) contain GABA. The somata of these fibers are located in the inferior-median protocerebrum (arrows in A and D) and in the inferior lateral protocerebrum (arrowhead in A). C and E: Sagittal sections through the central body. Staining in the CBU is restricted to layer II, while the other layers are devoid of GABA. The CBL is densely innervated by GABA-containing neurites. GABA positive fibers enter the CB via the posterior groove. D: Frontal section through one lateral accessory lobe (LAL). Fibers originating from cells in the inferior median protocerebrum run through the isthmus tract (indicated by asterisk) before they enter the CB. Knob-like staining is observed in the lateral triangle (double arrowheads). Additional intensive immunoreactivity is present in the median olive (arrowhead). F: Frontal section through the protocerebral bridge (PB) reveals the absence of GABA from this neuropil. G: Schematic drawing of a sagittal section through the CB. Regions highlighted in gray contain GABA positive fibers (modified from [18], [21]. CBAL, anterior lip of the central body upper division; N, noduli. Scale bars = 100 µm in A; 50 µm in B; 40 µm in C; 20 µm in D, E and F.

The CX of *Ch. biguttulus* is strongly innervated by bilateral pairs of GABA immunoreactive tangential neurons, with their somata in the inferior median protocerebrum (arrows in Fig. 3A and D). Additionally, a small number of neurons are located more laterally at the border to the inferior lateral protocerebrum (arrowhead in Fig. 3A). The entire lower division of the central body is densely innervated with GABAergic arborizations (Fig. 3A, B, C and E), while in the upper division only layer II contains sparse GABAergic neurites (Fig. 3B, C). The fibers of these neurons run through the isthmus tract. Sidebranches with knob-like appearence were detected in the lateral triangle and the median olive of the LALs (Fig. 3D). No GABA immunoreactive neurites could be detected in the protocerebral bridge (Fig. 3A and F) and the paired noduli (Fig. 3C).

### Citrulline immunoreactivity in the central complex

**Figure 4:**
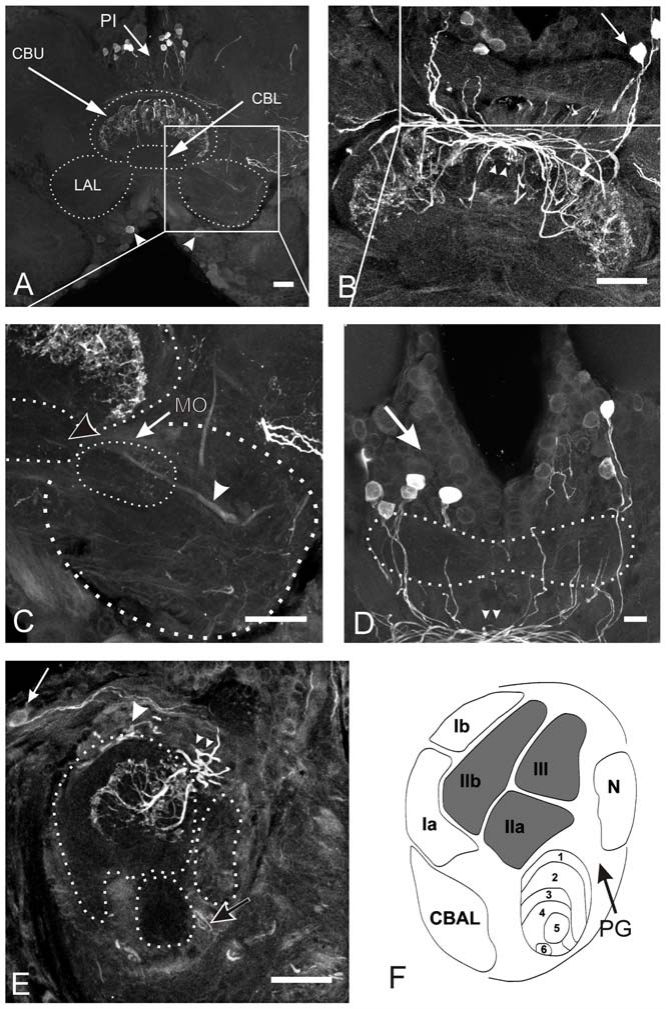
Citrulline immunoreactivity in the central complex. A–D: Frontal sections through the midbrain and central complex. E: Sagittal section through the central complex. Groups of citrulline immunoreactive cell bodies are located in the pars intecerebralis (PI, white arrows in A, B, D and E) and in the inferior median protocerebrum (arrowheads in A). Citrulline immunoreactivity is restricted to the layers II and III of the upper division (CBU) of the central body, while layer I and the entire lower division (CBL) was completely free of immunostaining. Two types of immunoreactive neurons were distinguished. Pontine neurons contributing to intensive labeling in the posterior chiasm (small arrowheads in B, D and E) and additional faint labeling in tracts entering the CB through the dorsal and posterior face (large arrowhead in E). Immunostaining in the posterior groove (black arrow in E) resulted from tangential neurons. The lateral accessory lobes (LAL) contained weak staining. Citrulline-ir was detected in the median olive (MO in C) and the ventral shell (white arrowhead in C) of the LAL. F: Schematic drawing of a sagittal section through the CB (modified from [18], [21]. Regions highlighted in gray contain citrulline positive fibers. CBAL = anterior lip of the central body, N = noduli. Scale bars = 50 µm in A, B and C; 20 µm in D and E.

Citrulline is generated as a side product during the formation of NO and its accumulation in neurons is regarded as a correlate for recent activity connected to NO release [11], [27]. Anti-citrulline immunocytochemistry in *Ch. biguttulus* brains labeled subsets of nitric oxide synthase-expressing and NADPH diaphorase-positive neurons that have previously been described in the locust *S. gregaria* [36] and in *Ch. biguttulus* [10]. Citrulline immunoreactive fibers emerged from somata in the anterior pars intercerebralis (arrows in Fig. 4A, B, D and E) and the ventro-median protocerebrum (arrowheads in Fig. 4A) to innervate the upper division of the central body (Fig. 4A, B and E). All other central complex neuropils were entirely free of citrulline-associated labeling. Sagittal sections (Fig. 4E) revealed that citrulline accumulation was restricted to layers II and III of the upper division, whereas layer I contained no detectable immunofluorescence. Citrulline immunopositive neurons included mainly pontine and probably also tangential neurons. Fibers of pontine neurons have their somata in the anterior PI. Their fibers run posteriorly towards the PB but do not innervate the PB (Fig. 4B, D and E) and instead turn anteriorly and enter the CB through the posterior chiasm (small arrowheads in Fig. 4B, D and E) to innervate columns of other layers and enter the central body through the posterior face ([37]; arrowheads in 5E). We were not able to distinguish whether individual neurons innervate only specific parts of layers II and III or both layers entirely. Citrulline immunoreactive tangential neurons entered the CX within the tract IT2 (arrowhead in Fig. 4C) and through the posterior groove (black arrow in Fig. 4E). These fibers also seemed to innervate the upper division of the CB. In line with earlier studies that described the distribution of nitric oxide synthase [10], [36], we were also able to detect a bilateral pair of immunoreactive somata in the ventro median protocerebrum (arrowheads in Fig. 4A) which was described as TL-1 neuron by Kurylas et al. [36]. Although these fibers have been demonstrated to innervate the CBL but not the CBU, no citrulline immunoreactivity was detected in the CBL in *Ch. biguttulus*. This raises the question whether these neurons innervate the CBU in *Ch. biguttulus* or whether our method was not sensitive enough to detect citrulline in the CBL. Citrulline-IR-fibers of unknown origin (either from tangential or from columnar neurons) left the CX via the IT1-tract (black arrowhead in Fig. 4C) and formed arborizations in the median olive of the lateral accessory lobes (Fig. 4C). This differs from the structures described in *S. gregaria* [36], where the median olive was free of NO-producing fibers.

### NO stimulated accumulation of cGMP in the central complex

**Figure 5:**
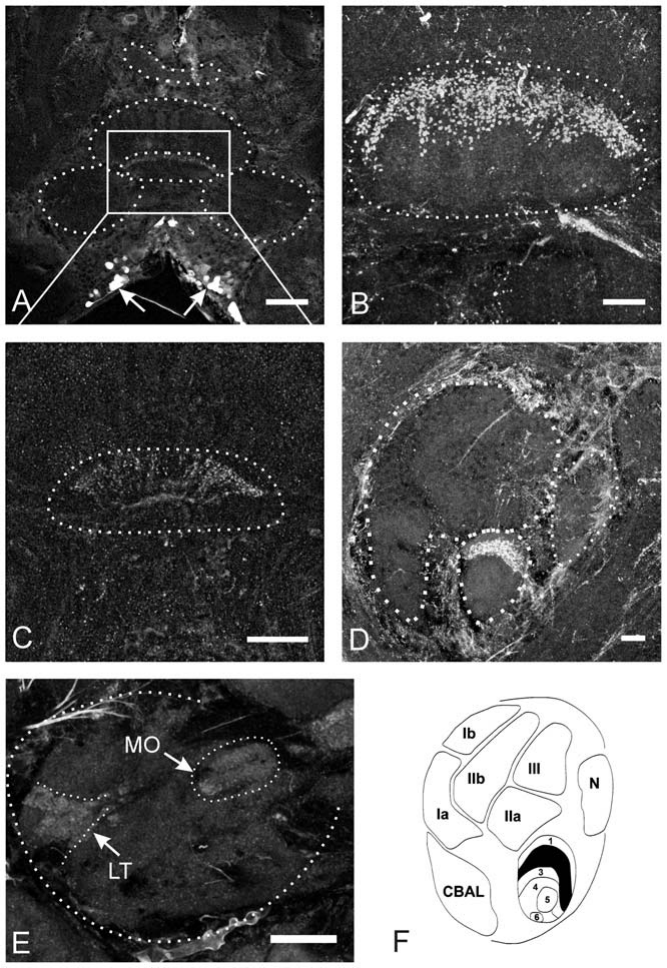
Nitric oxide-stimulated cGMP immunoreactivity in the central complex. A: Frontal section, through the central complex and surrounding midbrain structures. Within the central complex cGMP-ir is restricted to a particular layer of the CBL. Labeled cell bodies are located in the inferior median protocerebrum (indicated by arrows). B and C: Frontal sections through the CBL. Strong cGMP-ir is associated with fibers of tangential neurons projecting close to the anterior border of the CBL. The neurites enter the CBL from posterior direction and innervate the CBL in a fan-shaped fashion (best seen in C). Immunostaining in the CBL appears to be beaded (best seen in B and D) suggesting that labeled neurites represent presynaptic structures. D: Sagittal section through the central body: Staining in the CBL is restricted to layer II, while the other layers of the CBL and the entire CBU are completely devoid of staining (compare with F). E: Frontal section through one lateral accessory lobe (LAL) containing faint labeling in the lateral triangle (LT) and the median olive (MO). F: Schematic drawing of a sagittal section through the CB (modified from [18], [21]. Regions labeled black contain cGMP immunopositive neurites. CBAL = anterior lip of the central body, N = noduli. Scale bars = 100 µm in A; 20 µm in B, D and E; 10 µm in C.

Cyclic GMP immunoreactivity in the central complex (Fig. 5) was exclusively observed in tangential neurons innervating the lower division of the central body (Fig. 5A, B, C and D). Immunoreactive cell bodies were located in the infero-median protocerebrum (arrows in Fig. 5A). Sagittal sections revealed that accumulation of cGMP was resticted to neurites in layer 2 of the CBL (Fig. 5D, F). Labeling in the LAL (Fig. 5E) was generally weak but faint immunostaining appeared in both the median olive and the lateral triangle. Few preparations contained very faint NO-stimulated cGMP immunoreactivity in sparse fibers of the upper division (see also [10]), representing the exclusive source of NO in the central complex (see above). Given that staining in the CBU was very faint and was not present in every sample, whereas cGMP could be robustly detected in the CBU in every sample, we conclude that cGMP in the CBU is unlikely to play a major part in the control of sound production. Apart from this, no immunoreactivity could be detected in central complex neuropiles outside the CBL and brain regions surrounding the central complex were also free of NO-stimulated cGMP accumulation.

### GABA immunoreactive fibers accumulate cGMP upon NO stimulation

**Figure 6:**
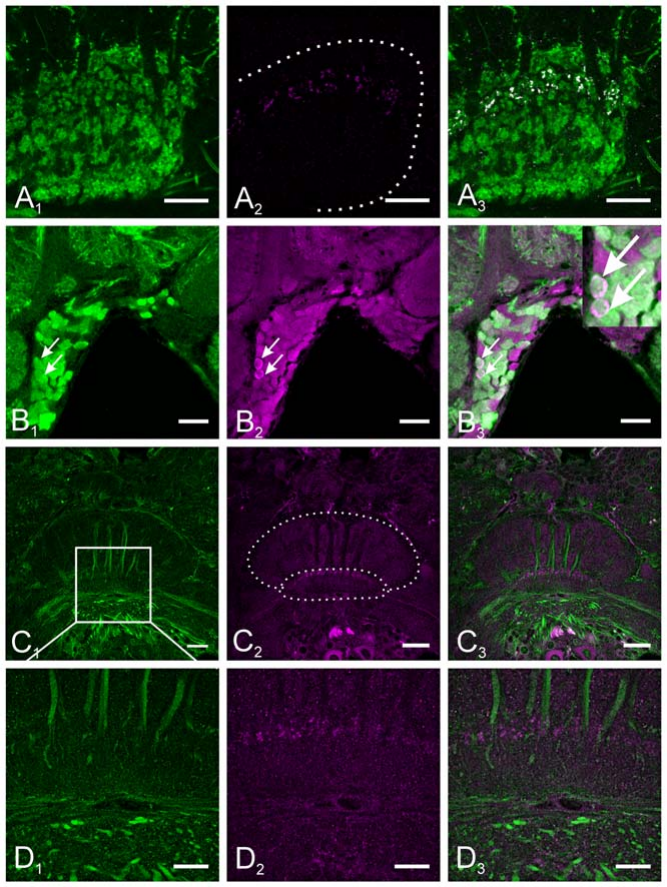
GABA, mAChR and NO-stimulated cGMP immunoreactivity in the central complex. A_1_–B_3_ Double labeling of GABA (green) and cGMP (magenta) in frontal sections through the midbrain. Colocalization (highlighted in white) was detected in tangential neurons of the central body lower division. All cGMP-immunoreactive neurites also contain GABA immunoreactivity, whereas only a subset of GABA-immunoreactive neurites accumulated detectable amounts of cGMP upon nitric oxide stimulation. Colocalisation of GABA and cGMP immunoreactivity was detected in five cell bodies per hemisphere in the infero-median protocerebrum (two of them are marked by arrows in B_1_–B_3_). C_1_–D_3_ Double labeling of mAChR (green) and cGMP (magenta) on frontal sections through the central complex. No colocalisation was detected, indicating that NO has no direct effect on mAChR-expressing columnar neurons of the central complex. Scale bars = 50 µm in B_1_–C_3_; 20 µm D_1_–D_3_; 10 µm in A_1_–A_3_.

GABA, NO donors and membrane permeable analogs of cGMP have been demonstrated to suppress grasshopper sound production [6], [10], [11]. Since both GABA and cGMP were detected in tangential neurons that innervate similar regions of the lower division of the central body we investigated the possibility of their coexpression by central complex neurons (Fig. 6A_1_–B_3_). Double labeling experiments demonstrated that NO-stimulated accumulation of cGMP occurred in GABAergic neurons of the CBL. Distance-based colocalisation analysis revealed that cGMP was primarily upregulated in GABAergic fibers (Fig. 6A_1_–A_3_), since 96% of cGMP positive fibers were also immunopositive for GABA. In contrast, only a subset of GABAergic fibers (21%) innervating the CBL accumulated detectable amounts of cGMP. Fibers that contained both, cGMP and GABA immunoreactivity were restricted to a dorsal layer of the CBL, presumably layer 2. Analysis for colocalization was focused on fibers in the CBL, since cGMP staining of the somata varied greatly between different preparations, while labeling of the fibers was similar within individual and among different preparations. Nevertheless, colocalisation of GABA and NO-stimulated cGMP was detected in somata located in the inferior-median protocerebrum (Fig. 6B_1_–B_3_), the region where somata of tangential neurons that innervate the lower division of the CB are localized.

### mAChR- expressing columnar neurons are not NO responsive

Activation of the NO/cGMP signaling pathway in the central complex has been demonstrated to suppress muscarine-stimulated sound production in restrained *Ch. biguttulus* [10]. Since both, mAChR-expressing columnar neurons and cGMP-accumulating tangential neurons extensively arborize in the lower division of the central body, we investigated the possibility that NO might stimulate the production of cGMP in these columnar output neurons of the central complex. Double labeling of mAChRs and NO-stimulated cGMP revealed no evidence for colocalization of the two antigens, although both were expressed in closely associated neurites in the lower division of the central body (Fig. 6C_1_–D_3_).

### GABA signaling is required for NO-mediated suppression of sound production

**Figure 7:**
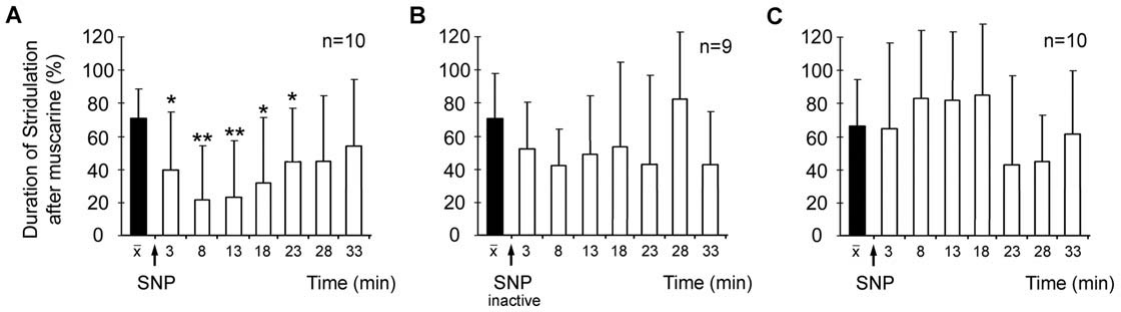
Pharmacological stimulation of sound production by pressure injection of neuroactive substances into the central complex of restrained male *Ch. biguttulus*. A: The duration of stridulation stimulated by 10^−4^ M muscarine (black column) was reversibly reduced by a single injection of the NO donor SNP (10^−3^ M) to the same site in the central complex. Significant differences (**: p≤0.01; *: p≤0.05) were calculated with respect to the muscarine pulses before SNP application. B: After overnight storage in an unsealed vessel, SNP solution loses its potency to generate NO upon injection into the central complex. Inactivated SNP had no inhibitory effect on muscarine-stimulated stridulation. C: The duration of stridulation stimulated by 10^−3^ M picrotoxin was unaffected by co-application of SNP (10^−3^ M) to the same site in the central complex.

Sound production in restrained *Ch. biguttulus* can be repeatedly stimulated by injections of identical small volumes of muscarine into the central body. A single injection of the NO donor SNP reversibly reduced the duration of sound production elicited by the standard muscarine pulses applied to the same site within the central complex (Fig. 7A; compare [10], [11]. The inhibitory effect remained absent when inactive SNP solution, which had been stored overnight in an unsealed vessel, was used in the same way (Fig. 7B), indicating that it was indeed the NO that mediated the suppression of sound production. Sound production in *Ch. biguttulus* can also be stimulated by injection of the chloride channel blocker picrotoxin [6] and the GABA depleting agent 3-mercaptopropionic acid (MPA; inhibits glutamate decarboxylase and activates GABA transaminase) into he central complex (unpublished own results), indicating a tonic release of GABA in the central complex in this experimental situation. Since immunocytochemical data (see above) suggested a functional coupling of NO- and GABA-mediated inhibitory signaling in the central complex, we investigated whether NO can suppress sound production while GABA signaling is inactivated. Sound production in restrained male *Ch. biguttulus* was repeatedly stimulated by regular series of identical injections of picrotoxin (10^−3^ M) to a fixed site in the central complex. After a few initial stimulations, each following picrotoxin stimulus elicited a similar duration of sound production. A single injection of the NO donor SNP was applied to the same site in the central complex and its influence on subsequent picrotoxin-stimulated sound production evaluated. In contrast to muscarine-stimulated sound production (Fig. 7A) no SNP-mediated reduction of picrotoxin-stimulated sound production was observed (Fig. 7C).

## Discussion

Recent studies implicated that the central complex processes spatial information [20], [38]–[40] and functions as a pre-motor control center in the insect brain (reviewed by [14], [41]–[44]. In particular, neural substrates that select and initiate certain rhythmic behaviors consisting of stereotype series of patterns (e.g. stridulation, wing vibration, walking, turning, gap crossing) seem to reside in the central complex neuropils [9], [45]–[47]. In acoustically communicating grasshoppers the central complex selects and coordinates the production of situation-dependent song types. Pharmacological treatments that increased excitation or decreased inhibition both stimulated sound production and demonstrated that this behavior is controlled by a balance of excitation and inhibition within the central complex [5], [6], [9]. Until now several neurotransmitters and neuromodulators have been identified that promote (ACh: [5], [12]; proctolin, dopamine: own unpublished results) or suppress (NO/cGMP: [10], [11]; GABA: [6]) both, spontaneous and conspecific song-stimulated stridulation.

### mAChRs the central complex

Activation of mAChRs in the central complex of grasshoppers has been shown to stimulate sound production by activation of both, adenylyl cyclase- and phospholipase C- initiated second messenger pathways [8], [9]. Immunocytochemistry with an antibody raised against a *D. melanogaster* mAChR [23], whose specificity for *Ch.biguttulus* has been proven by western blotting [12] and by pharmacological in vitro studies [48], labeled two types of columnar neurons (Fig. 2). One type innervated the lower division of the central body, while the other projected directly from the upper division into the contralateral lateral accessory lobe bypassing the lower division along its anterior border. Muscarinic receptor-expressing columnar neurons with arborizations in the CBL resembled the CL1-fiber type described in the locust *S. gregaria* [21]. Since muscarinic excitation in the central body promotes sound production in response to acoustic stimulation with the song of conspecific females [8], [12], we speculate that this type of mAChR-expressing columnar neurons most likely receives auditory sensory input. Electrophysiological recordings will be necessary to validate this assumption. The other type of mAChR-expressing columnar neuron projects through layer I of the upper division and lacks any arborizations in the CBL. Neurons with similar anatomical features, including similar position of cell bodies have been described in *S. gregaria* by Homberg and coworkers [49]. These CP-neurons are sensitive to polarized light. In *S. gregaria*, the neurons restrict their dendritic arborizations to single columns of the protocerebral bridge and send axonal projections through the CB to the contralateral LAL. Since they have no ramifications in the central body, it seems unlikely, that these neurons are activated by injections of muscarine.

Since columnar neurons are the presumed output neurons of the central complex that, among other targets, connect to premotor interneurons in the lateral accessory lobes, the mAChR-expressing columnar neurons may provide the CX output that, either directly or via another set of interneurons, activate the descending stridulatory command neurons.

Presently, we have no information about the origin of cholinergic input that excites the mAChR-expressing columnar neurons in behavioral situations that favor stridulation. Acetylcholine-esterase staining provided strong labeling in the lower division of the CB [12], but this method stains only synaptic regions and with low anatomical resolution and thus does not provide information about cell body locations and axonal projections. Assuming that cholinergic input to the CBL is mediated via tangential neurons we speculate that cholinergic input arises from the LAL, since this region is described as the only input region to the CBL [21] and also contained strong ACh-esterase activity [12].

### GABA in the central complex

GABA is the major fast inhibitory transmitter in the insect brain. Injections of GABA into the central complex of grasshoppers inhibit stridulation induced by cholinergic agonists after short latency and with short duration [6] suggesting that GABA_A_ receptors mediate this effect. Outside periods of stridulation, sound production seems to be suppressed by tonic release of GABA in the CX since both blocking ionotropic chloride channel-associated receptors with picrotoxin and inhibition of GABA production with 3-mercaptopropionic acid (unpublished own results) are sufficient to release stridulation by disinhibition. The distribution of GABA in the CX of *Ch. biguttulus* (Fig. 3) is very similar to that of other insect species [14], [17], [50]–[55] implicating that apart from its specific function on sound production the general functional role of GABA in information processing and motor control in the CX may also be conserved. The CX is strongly innervated by tangential neurons having their somata in the inferior median and inferior lateral protocerebrum. The entire lower division of the central body is densely supplied with GABAergic fibers, while only parts of the CBU, namely layer II, also contain GABAergic neurites. This staining pattern is virtually the same as in *S. gregaria*, with the exception that in *Ch. biguttulus* only layer II of the CBU is supplied with GABAergic fibers (Fig. 3B) and not also layer I [55]. On the basis of soma position and projection patterns in the CBL, Müller et al. [21] distinguished five different types of tangential neurons in *S. gregaria* that innervated the lower division. In comparison to that study, GABA immunoreactive neurons of *Ch. biguttulus* most likely belong to the types TL2, TL3 and TL4. The two other types of tangential neurons described in that study (TL1, TL5) had their somata in the ventro-median protocerebrum and the pars intercerebralis (PI) where no GABA immunoreactive cell bodies were detected in *Ch. biguttulus*.

### The NO-cGMP signaling pathway

NO has been shown to inhibit muscarine-stimulated stridulation, when injected into the central body at the same site as muscarine. This inhibitory effect is mediated by activation of soluble guanylate-cyclase and subsequent increase of cytosolic cGMP levels [10]. Antisera against citrulline (a side product during the formation of NO) and cGMP have been shown to serve as valuable tools to label the activation status of neurons that actively produce (citrulline) or respond to NO (cGMP) in various vertebrate and invertebrate preparations [24], [27], [56]–[59].

#### Citrulline in the central complex

NO-producing neurites in the central complex of *Ch. biguttulus* have previously been labeled by NADPHdiaphorase histochemistry and uNOS-immunocytochemistry [10]. Anti-citrulline immunocytochemistry labeled a subset of neurons described in these earlier studies [10], [11, this study] and similar results with these three markers for NO-producing neurons have been obtained in the CX of locusts [27], [36]. Citrulline accumulation in NOS-expressing central complex neurons reflects activation of these neurons prior to brain fixation. Being restrained, as it is necessary for brain dissection, is a situation that is unfavorable for sound production and hence sound production might be suppressed by NO release in the central body leading to the accumulation of citrulline in these neurons. Representation of unfavorable situations for sound production by NO release in the upper division of the central body was recently supported by pharmaco-behavioral studies with *Ch. biguttulus* females. Systemic application of the NOS inhibitor aminoguanidine caused both, a substantial reduction of citrulline accumulation in the central complex and an increased responsiveness of female grasshoppers to males' calling songs [11]. In contrast to these CX neurons, NOS-expressing interneurons in the antennal lobes did not accumulate citrulline, indicating that they were not activated in this experimental situation (similar findings in locusts by [27]). Citrulline was exclusively detected in layers II and III of the CBU of *Ch. biguttulus*. In contrast, previous studies on *S. gregaria* [27] found citrulline-immunoreactive arborizations in all neuropils of the CX and distinguished at least six types of neurons that innervated at least one portion of the CX. In addition, citrulline was also detected in the lower division of another locust species, *Locusta migratoria* [60]. Though we used the same antibody as Siegl and colleagues and stainings were repeated by different members of our lab with slightly differing protocols, citrulline immunoreactivity was only detected in pontine neurons and probably also in some tangential neurons but was always absent from the CBL. These findings are in line with the distribution of NOS expressing neurons described in previous studies on *Ch. biguttulus* [10].

#### Identification of cGMP accumulating neurites in the CBL

NO-stimulated accumulation of cGMP in the central complex (enhanced by YC-1 treatment and phosphodiesterase inhibition) was exclusively detected in GABA-containing tangential neurons innervating layer 2 of the CBL. Consequently, these fibers represent the only candidate targets for NO released within the central body of *Ch. biguttulus*. Cyclic GMP immunopositive neurites belonged to neurons that appeared similar to TL2 and TL3 neurons described in *S. gregaria* by Müller and coworkers [21] and TL2 neurons of this species have been reported to accumulate cGMP following stimulation with NO [27]. These tangential neurons are suggested to provide input from LALs to the lower division of the central body. In contrast to *Ch. biguttulus*, additional NO-responsive neurons were detected in the CBU, the noduli and the LAL of *S. gregaria* and *L. migratoria* [27] indicating differences between these grasshopper species in both the NO-producing and NO-responding neurons of the CX. Muscarinic AChR-expressing columnar neurons were excluded as potential targets of NO, since they did not accumulate cGMP following NO stimulation. In contrast, essentially all NO-stimulated cGMP accumulation within the entire central body and the LAL, occurred in GABA-containing tangential neurons that innervated layer II of the CBL. Tangential neurons are suggested to receive synaptic input in the LAL and release transmitter from beaded arborizations in the CBL or other CX compartments [21]. Colocalization of NO-sensitive soluble guanylyl cyclase or NO-stimulated cGMP with GABA has previously been described in the antennal lobes of locusts and moths [56], [61] and potentiation of presynaptic GABA release by cGMP-initiated processes has been demonstrated in rat brain [62]–[64], rat spinal cord [65], turtle retina [66] and lamprey spinal cord [67]. In insects NO-mediated transmission causing the formation of cGMP has previously been reported at the neuromuscular junction, in sensory neuropils processing visual, olfactory and various mechanosensory information (reviews: [68], [69]) and in the mushroom bodies [70]. In these systems sources and targets of NO are located in close proximity. In the central complex of *Ch. biguttulus*, NO is exclusively produced in the CBU, which has been confirmed by three different methods to label NO-producing cells (NADPHdiaphorase histochemistry, anti-NOS and anti-citrulline immunocytochemistry). In contrast, NO-responsive neurites are exclusively located in the CBL at an approximate distance of 40–60 µm from NO production sites. This distance lies well within the action range of NO that was estimated in other preparations ([71]: 90 µm; [72]: 300 µm; [73]: 100 µm; [74]: >200 µm) and thus supports our hypothesis of a NO-mediated direct information flow between the CBU and the CBL. A direct information transfer between the CBU and the CBL via connecting neuronal projections could so far only be detected in *D. melanogaster* [17] while histological investigations in *S. gregaria* revealed no tracts that could mediate direct exchange of information between the two central body subdivisions [22].

Further support for the hypothesis that NO-responsive GABAergic tangential neurons could mediate the inhibitory effects of endogenous NO release in the central complex arises from pharmacological experiments in this and previous studies. In the present study, the NO donor SNP suppressed muscarine-stimulated sound production when injected to the same site in the central complex where muscarine elicited singing. In a series of similar experiments, SNP could not suppress stridulation that was elicited by picrotoxin-mediated inhibition of GABA_A_ receptors. In both experiments, the injection capillary must have been placed in the CBL, since dendrites of muscarinic AChR-expressing neurons overlap with presynaptic terminals of GABAergic tangential neurons in this neuropil. A number of previous experiments in which substances with limited diffusion radius were injected to sites where muscarine stimulated sound production are also in line with this result. Muscarine-stimulated sound production was reversibly suppressed by 8-Br-cGMP and the inhibitor of cGMP-dependent phosphodiesterase Zaprinast [10], suggesting that their sites of action are very closely located to the dendrites of the muscarinic AChR expressing columnar neurons.

### Information processing in the CBL

**Figure 8:**
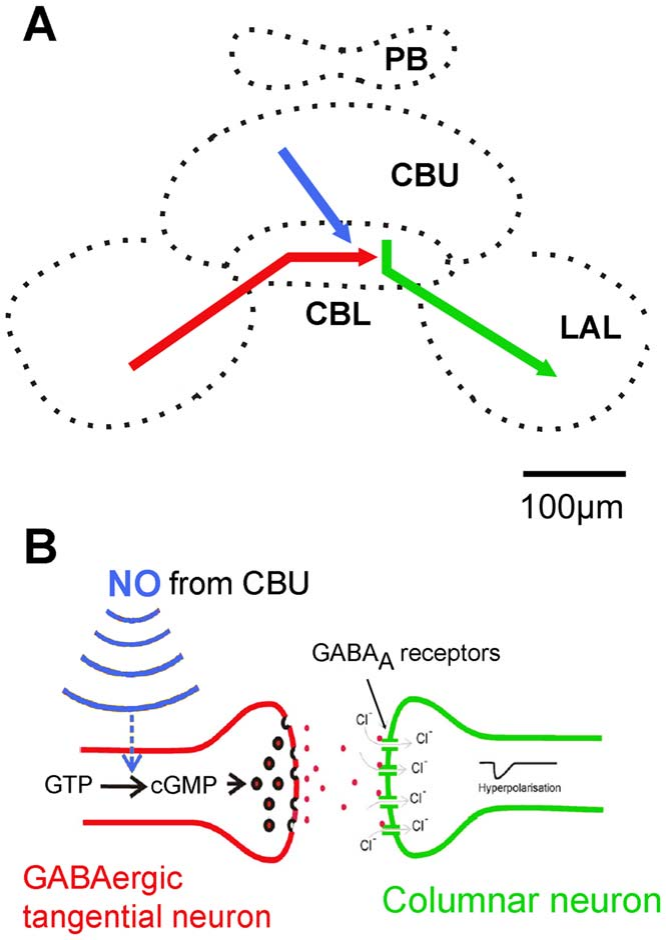
Information flow in the central body with respect to the control of grasshopper sound production. Each type of neuron included represents bilateral groups of several cells. A: Columnar neurons (green) are activated by both nicotinic and muscarinic input from unknown presynaptic neurons. Sufficient cumulative activity of columnar neurons initiates stridulation via direct or indirect activation of descending stridulatory command neurons. Tangential neurons release GABA in the lower division of the central body (CBL) that most likely directly inhibits columnar neurons through picrotoxin-sensitive ionotropic GABA_A_ receptors. Part of the synaptic release of GABA in the CBL is potentiated by nitric oxide (NO) released in the upper division (CBL). B: Three transmitters known to promote (ACh) or suppress (GABA and NO) grasshopper sound production converge on GABAergic synapses in the CBL. NO released from neurites in the CBU activates soluble guanylate cyclase (sGC) in a subpopulation of GABAergic tangential neurons in the CBL. Accumulation of cGMP potentiates GABA release from these tangential cells. The balance of activating and inhibiting inputs to these columnar neurons determines whether sound production will be executed.

Our results indicate that particularly the lower division of the central body may play an important role for the initiation of sound production in *Ch. biguttulus* since various signaling pathways known to promote (ACh) or to suppress (GABA and NO-stimulated cGMP) sound production converge in this neuropil (Fig. 8). All three pharmacological signals converge in the dendritic regions of mAChR-expressing columnar output neurons of the central complex that seem to play a decisive role for the initiation of grasshopper sound production. The motivation to sing may profoundly depend on the activity of this cell population, which is increased by the level of second messengers that accumulate upon stimulation of their muscarinic receptors and potentially decreased by the release of GABA from tangential neurons. Previous studies [8], [9] demonstrated that muscarinic excitation is mediated by activation of both the phospholipase C and the adenylyl cyclase second messenger pathways. Whether both pathways may be expressed in the same or different columnar neurons and whether both pathways are activated by the same or different types of mAChR is still unknown. The antibody used in this study was raised against a *D. melanogaster* mAChR, which until today remains the only molecularly identifed muscarinic receptor in insects [23]. Studies on frog oocytes, Cos-7 and S2-cell lines that heterologously expressed this receptor demonstrated its positive coupling to the phospholipase C signaling pathway [23], [75]. Since data from various pharmacological studies indicated that insects express multiple types of pre- and postsynaptic mAChRs that, alternatively to activating phospholipase C, can activate or inhibit adenylyl cyclase-dependent second messenger cascades [9], [76], expression of a second type of mAChR in the central complex that is not detected by this antibody cannot be excluded.

Various studies on locusts and other insects implicated that the central complex is involved in the processing of spatial information [22], [38], [39].

In a recent paper it was shown, that single columns of the protocerebral bridge respond to specific e-vector orientations of dorsally presented polarized light, resulting in a maplike representation of this visual feature [39]. Work from our lab on the species *Ch. biguttulus* indicated that recognition of conspecific song activates cholinergic projections into the central complex [8], [12]. Both types of stimuli, polarized light and species-specific acoustic stimuli, are processed in the central complex by similar types of columnar neurons. Whether individual neurons of this type process both visual and acoustic sensory stimuli or different subgroups exist, that process either one or the other stimulus remains to be shown. Multimodal functions of columnar neurons would be conceivable since both stimuli (polarized light and acoustic signals) are used for orientation [77]–[79]. Desert locusts use the polarization pattern in the sky for spatial navigation during their migratory phases, while grasshoppers perform sound based localization of potential mating partners. It is tempting to speculate that columnar neurons in the central complex of acoustically communicating grasshoppers may encode the position of a calling reproductive partner.

In contrast, unfavorable situations that suppress sound production, like being restrained or being handled by the experimentor seem to activate NO release in the upper division of the CB from a group of pars intercerebralis neurons that expressed strong citrulline immunoreactivity. The hypothesis that unfavorable situations are represented through NO release in the central complex is supported by results from pharmaco-behavioral studies with *Ch. biguttulus* females. Systemic application of the NOS inhibitor aminoguanidine prevented the accumulation of citrulline in the central body upper division and enhanced sound production in response to acoustic stimulation with the male calling song [11]. NO-stimulated cGMP accumulation has been demonstrated to increase GABAergic transmission in various central nervous neuropils [63]–[67]. As demonstrated in these studies, the highly diffusible volume signal NO may also affect a large portion of the subgroup of GABA expressing tangential neurons in the grasshopper central complex, leading to inhibition of most columnar output neurons to effectively suppress stridulation in non-favorable situations.

By combining neuroanatomical data with the results of pharmaco-behavioral experiments from this and earlier studies, the present study reveals first insight into the flow of information related to the control of sound production within the central complex of acoustically communicating grasshoppers (summarizing scheme in Fig. 8). This crude framework for information flow in the should facilitate the examination of other signaling systems (dopamine, tyramine, various neuropeptides) that contribute to the control of grasshopper sound production. Co-labeling studies against components of different signaling pathways will refine our knowledge of information flow within the central complex.

## Supporting Information

Figure S1 Preadsorption study to demonstrate the specificity of the guinea-pig anti-GABA antiserum. The left panel (A) shows the results of staining experiments in which the primary antiserum has been preadsorbed with GABA while the right panel (B) shows the results of the positive control. Specific staining was entirely abolished through preincubation of the primary antisera with the 20 mg/ml GABA-BSA antigen in the central body (A_1_), the calyx of the mushroom bodies (A_2_) and the optic lobes (A_3_), three regions that showed high intense GABA-like-immunoreactivity in both, neurites and cell bodies (B). Scale bars: 50 µm in A_2_ and B_2_ and 100 µm in A_1_, A_3_, B_1_ and B_3_).
(TIF)

Figure S2 Muscarinic AChR immunoreactivity in the brain of *Ch. biguttulus* outside the central complex. Intensely labeled neurites were detected in the medulla of the optic lobes (A and B) and emerging from in the inner core of the antennal lobe (C). The antibody used in our study labeled the same brain regions in *D. melanogaster*. Immunoreactivity was also detected in the calyx (D) and the pedunculus (E) of the mushroom bodies of *Ch. biguttulus*. Though this has so far not been described for *D. melanogaster*, physiological studies on honeybees implicated a functional role for muscarinergic signaling in this brain region.
(TIF)
