# Supplementary figures and images for: Neurochemical Architecture of the Central Complex Related to Its Function in the Control of Grasshopper Acoustic Communication
Source: PLoS One. 2011 Sep 28;6(9):e25613. doi: 10.1371/journal.pone.0025613 (PMC3182233; doi:10.1371/journal.pone.0025613)

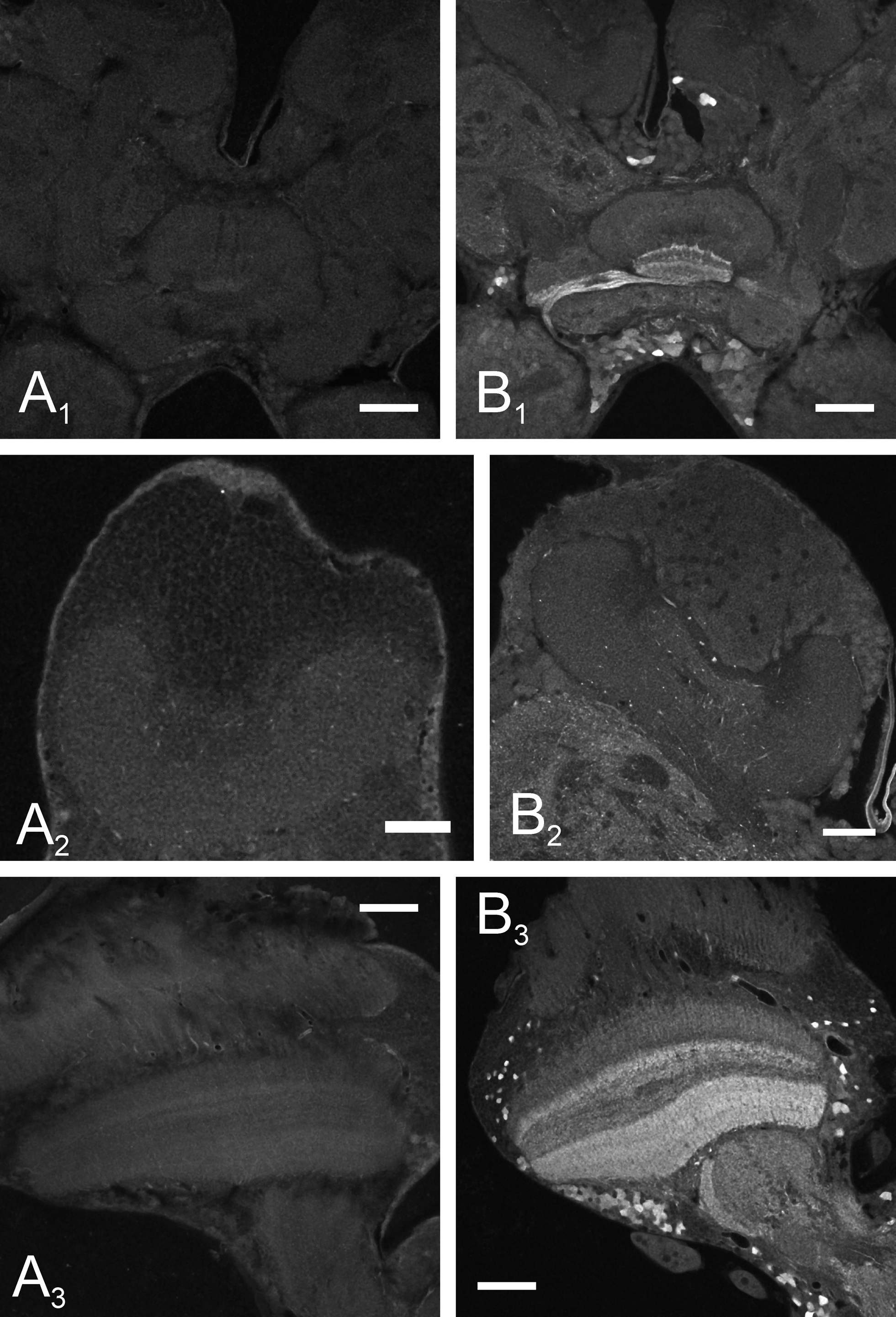

Supplement: Figure S1 — Preadsorption study to demonstrate the specificity of the guinea-pig anti-GABA antiserum. The left panel (A) shows the results of staining experiments in which the primary antiserum has been preadsorbed with GABA while the right panel (B) shows the results of the positive control. Specific staining was entirely abolished through preincubation of the primary antisera with the 20 mg/ml GABA-BSA antigen in the central body (A1), the calyx of the mushroom bodies (A2) and the optic lobes (A3), three regions that showed high intense GABA-like-immunoreactivity in both, neurites and cell bodies (B). Scale bars: 50 µm in A2 and B2 and 100 µm in A1, A3, B1 and B3). (TIF) [file pone.0025613.s001.tif]

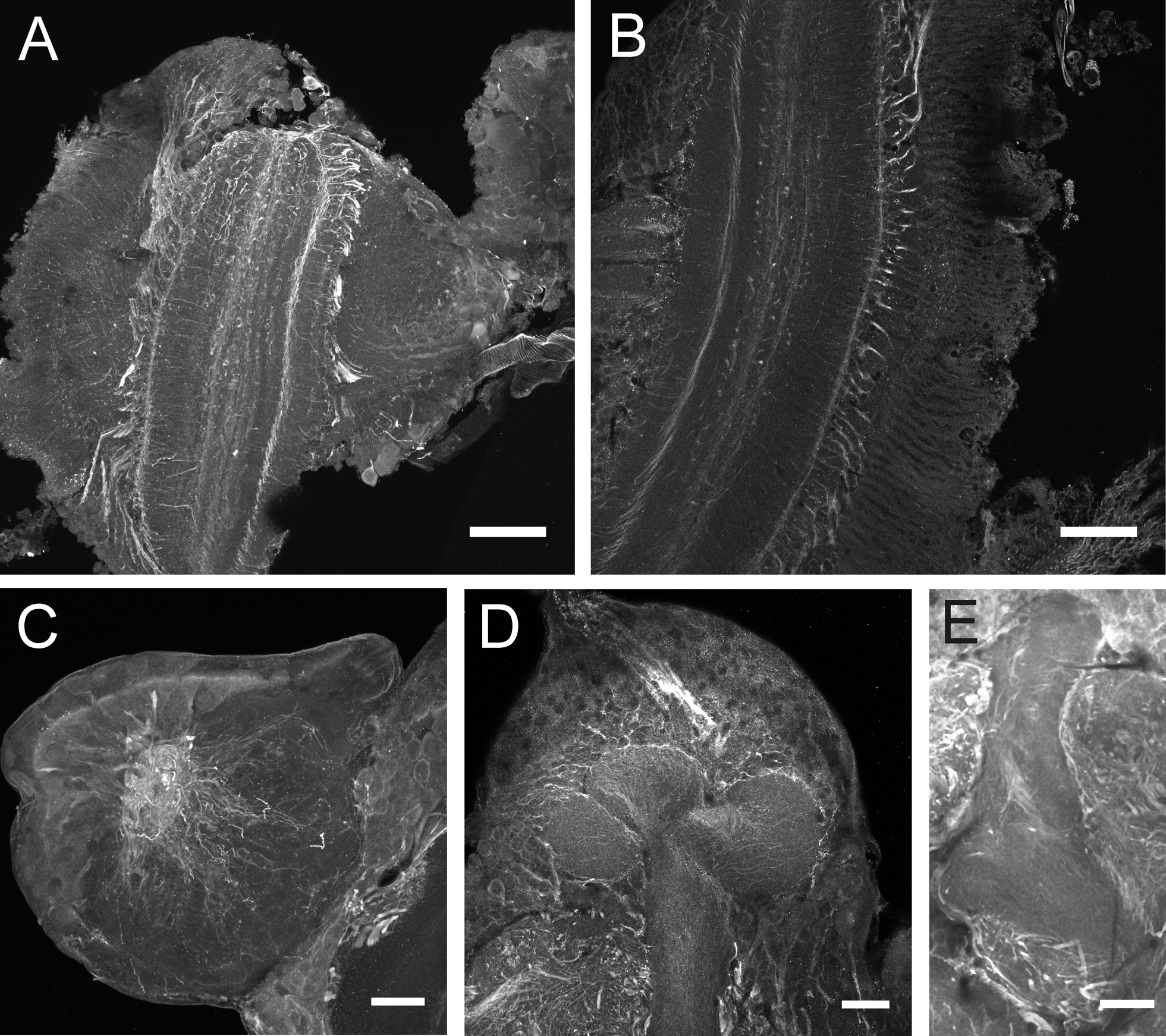

Supplement: Figure S2 — Muscarinic AChR immunoreactivity in the brain of Ch. biguttulus outside the central complex. Intensely labeled neurites were detected in the medulla of the optic lobes (A and B) and emerging from in the inner core of the antennal lobe (C). The antibody used in our study labeled the same brain regions in D. melanogaster. Immunoreactivity was also detected in the calyx (D) and the pedunculus (E) of the mushroom bodies of Ch. biguttulus. Though this has so far not been described for D. melanogaster, physiological studies on honeybees implicated a functional role for muscarinergic signaling in this brain region. (TIF) [file pone.0025613.s002.tif]
